# Transtuzumab induced organizing pneumonia: a case report

**DOI:** 10.1186/s40064-016-3647-6

**Published:** 2016-11-14

**Authors:** Ajay Gupta, Louise Teo, Philip Masel, David Godbolt, Geoffrey Beadle

**Affiliations:** 1 Asian Cancer Center, Faridabad, India; 2 Medical Oncology, Hervey Bay Hospital, Hervey Bay, QLD Australia; 3 Department of Thoracic Medicine, The Prince Charles Hospital, Chermside, QLD Australia; 4 Department of Pathology, The Prince Charles Hospital, Chermside, QLD Australia; 5 Department of Medical Oncology, Wesley Hospital, Auchenflower, QLD Australia

**Keywords:** Transtuzumab, Organizing pneumonia, Interstitial, Steroids, Lapatinib

## Abstract

**Background:**

Patients with metastatic breast cancer often have pulmonary symptoms with varying aetiologies. Transtuzumab is an extremely important drug used in the treatment of Her 2 neu over-expressing breast cancers. In this report we describe a case of organizing pneumonia associated with use of transtuzumab in metastatic breast cancer. Only three such cases have previously been reported.

**Case description:**

A 43 year old lady with Her 2 neu 3+, estrogen and progesterone receptor positive, metastatic breast cancer was started on weekly transtuzumab and albumen bound paclitaxel. She was admitted with an episode of bilateral pneumonitis after her fourth dose of therapy. It was managed conservatively with antibiotics. Subsequently, single agent transtuzumab was administered resulting in an anaphylactoid reaction followed by worsening dyspnoea requiring hospitalization and oxygen support for 3 days.

**Discussion and evaluation:**

The pneumonitis warranted further investigations including bronchoscopy which were performed. Bronchoscopic biopsy was consistent with a diagnosis of organizing pneumonia. Transtuzumab was stopped and she was started on steroids (dexamethasone). She improved remarkably and was discharged home on hormonal therapy, lapatinib and tapering doses of steroids.

**Conclusion:**

Organizing pneumonia closely mimics infection or progressive disease and can be difficult to diagnose in the setting of malignancy. Correct diagnosis is of paramount importance since delay in treatment can result in significantly adverse patient outcomes.

## Background

Patients with metastatic breast cancer often have pulmonary symptoms with varying aetiologies. Organizing pneumonia mimics infection or progressive malignancy and can be difficult to diagnose especially in the setting of malignancy. Transtuzumab is a monoclonal antibody against the Her-2 neu epidermal growth factor receptor, used in the treatment of Her 2 neu over-expressing breast cancers. Only three cases of organising pneumonia, in association with transtuzumab therapy, have previously been described (Travis et al. 2013; King and Mortenson 1992; Mokhtari et al. 2002; Cook-Burns 2001; Vahid and Mehrotra 2006; Perez et al. 2014; Pepels et al. 2009; Romond et al. 2005; Radzikowska et al. 2003; Taus-García et al. 2010; Grudny et al. 2004). We present the report of such a case.

## Case presentation

A 43-year-old, premenopausal, lady, was evaluated at an outside hospital for complaints of a lump in her right breast of three months duration. She also had severe bone pains and numbness in her chin, since the last one month. She was diagnosed to have metastatic breast cancer. Biopsy was suggestive of high grade infiltrating duct carcinoma which was further characterised as being estrogen, progesterone receptor positive and Her 2 neu 3+ by immunohistochemistry. Contrast Enhanced Computerised Tomography scans (CECT scans) of the chest and abdomen and Magnetic Resonance Imaging (MRI) of the brain, head and neck region revealed multiple lung, liver and bone metastases including the right acromion as well as parameningeal and right mandibular bony infiltration which was consistent with mental nerve neuropathy.

**Fig. 1:**
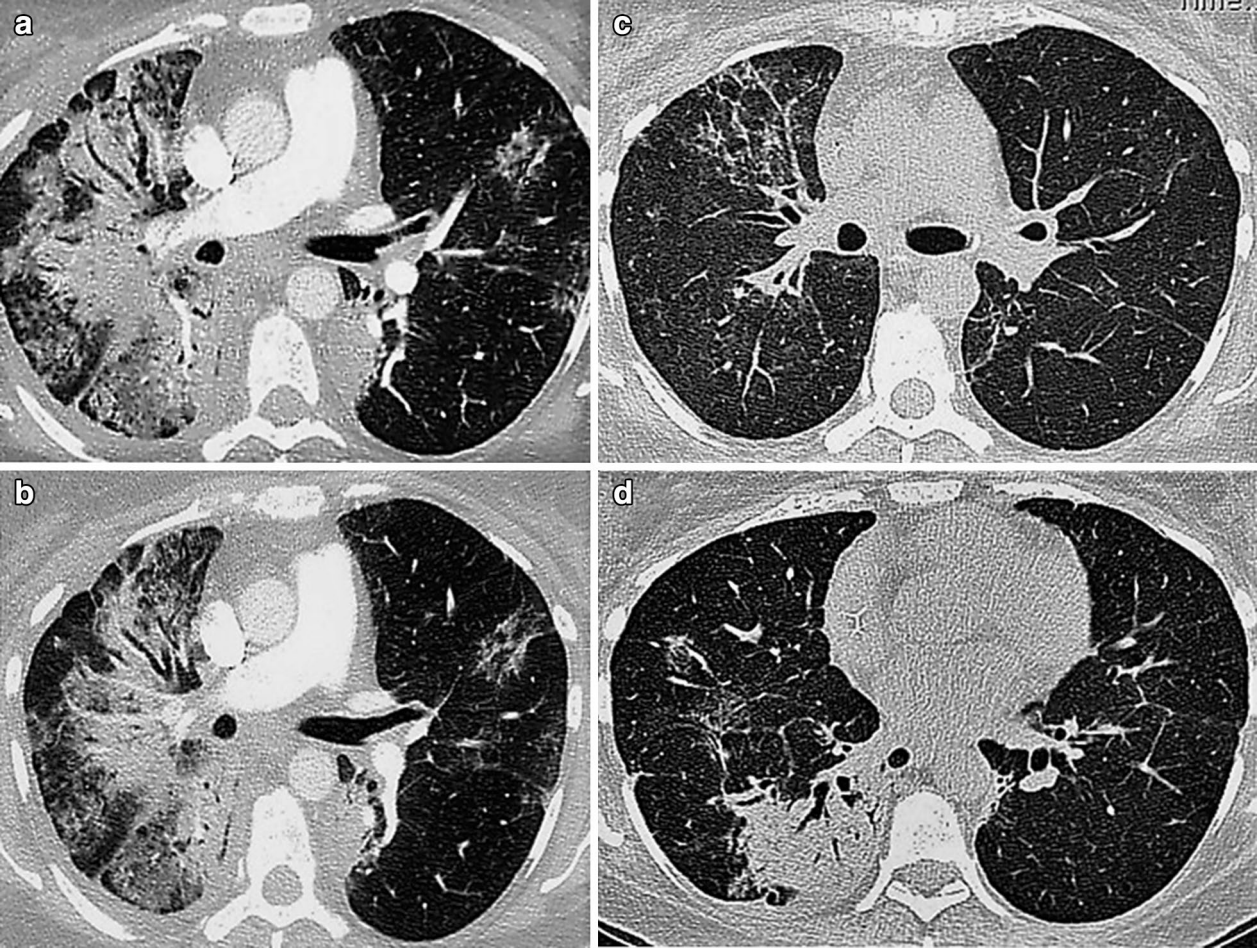
CECT of the Chest revealing. **a** Extensive right upper lobe consolidation, bronchocentric consolidation with air bronchogram, band like and peripheral consolidation and **b** the “reversed halo” sign of focal perilobular consolidation with central ground-glass opacities suggestive of organizing pneumonia. **c**, **d** CECT of the Chest before discharge revealing marked resolution of the organizing pneumonia after cessation of transtuzumab

She was given palliative radiation to the painful right acromial lesion. She was then started on chemotherapy with albumen bound paclitaxel 100 mg/m^2^ weekly and transtuzumab 4 mg/kg as loading dose followed by 2 mg/kg weekly. Two to four hours following each dose of transtuzumab, she would have episodes of high grade fever of up to 39 °C associated with mild chills, resolving spontaneously without intervention over the next 1–3 days. After the fourth dose, she developed bilateral pneumonitis (non-neutropenic) for which she had to be admitted to hospital for treatment with intravenous antibiotics. The CECT scan of the chest revealed extensive bilateral patchy areas of air space disease with interstitial involvement. Two weeks later, she improved and was given another dose of transtuzumab and albumen bound paclitaxel and then discharged. A week after receipt of her last dose, she presented to our hospital emergency with complaints of respiratory distress and hypoxia. CECT Scan of the chest and abdomen revealed dense extensive right upper and left lower lobe para-mediastinal consolidation along with smaller, bilateral patches and air bronchograms consistent with a diagnosis of organizing pneumonia (Fig. 1a, b). Septal lines and interstitial opacities in the mid lower zones suggested lymphangitis. There was evidence of progressive disease with multiple liver and bone metastases. The patient was started on oxygen, antibiotics, intravenous dexamethasone and supportive care. In view of progressive disease, albumen bound paclitaxel was discontinued. Hormonal therapy comprising goserelin depot preparation 3.6 mg administered subcutaneously every 28 days and anastrazole orally 1 mg daily was commenced.

Four days later, single agent transtuzumab was administered, to which she developed an anaphylactoid reaction with symptoms of acute breathlessness and hypotension. This was managed successfully with steroids, antihistaminics and intravenous fluids. The reaction resolved but her breathlessness did not and she in fact developed progressively worsening dyspnoea, with an oxygen saturation of 84% on room air, requiring supplemental oxygen, antibiotics and parenteral steroids. Her condition improved after 3 days of supportive care. Parenteral steroids were then stopped and oral dexamethasone 8 mg thrice daily was commenced. A bronchoscopy was conducted.

**Fig. 2:**
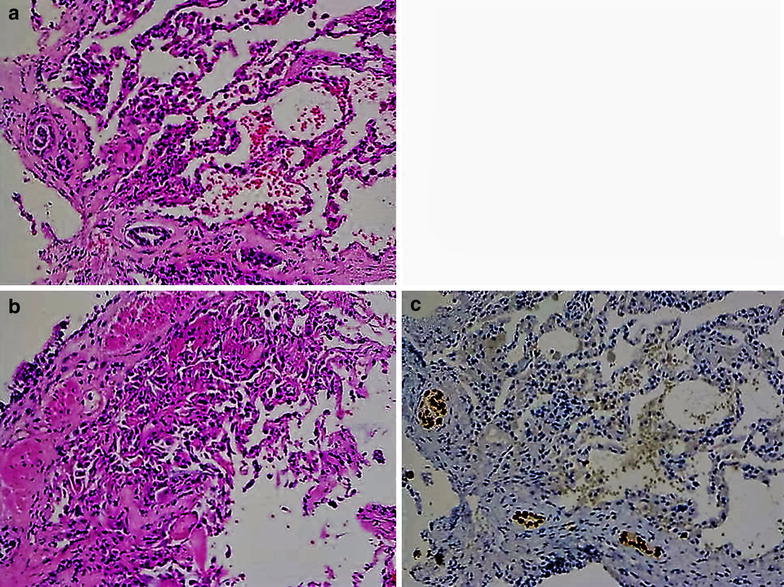
H&E staining 200× photomicrographs showing **a** focal intra-alveolar fibrin deposition with patchy reactive type II pneumocyte hyperplasia and focal organising pneumonia; **b** focal infiltrate of polygonal cells forming tubular like structures; **c** Described infiltrate of polygonal cells positive for GATA3 consistent with metastatic breast carcinoma

The biopsy revealed patchy intra-alveolar haemorrhage and fibrin deposition with collections of intra-alveolar foamy macrophages, patchy reactive type II pneumocyte hyperplasia and focal organizing pneumonia (Fig. 2a). There was also a localised interstitial infiltrate of polygonal cells forming tubular structures and a small ill-defined sheet (Fig. 2b). Changes suggestive of lymphatic invasion were evident. The polygonal cells were positive for estrogen receptor and GATA 3 (Fig. 2c) and negative for TTF1, consistent with metastatic breast carcinoma. There was no history of exposure to fumes and no microbiological or serological evidence of any pathogens or collagen-vascular disease.

Her symptoms resolved over the next two weeks and she was discharged home. The CECT scan of the chest done at discharge (Fig. 1c, d) revealed marked reduction in the organizing pneumonia and interstitial infiltrates. Lapatinib 1250 mg daily was added to her treatment regimen. Dexamethasone was slowly tapered off over the next 4 weeks. At last follow-up, 8 weeks post-discharge, she was doing well on therapy.

Organizing pneumonia or bronchiolitis obliterans organizing pneumonia (BOOP) is one of the major Idiopathic Interstitial Pneumonias (IIP) (Travis et al. 2013). Typical radiological features include multiple alveolar opacities, usually bilateral and peripheral, often migratory, varying in size from a few centimeters to a whole lobe, with air bronchograms often present in consolidated opacities: as in our case. The disease is pathologically defined by the presence of buds of granulation tissue formed by fibrin exudates, fibroblasts, myofibroblasts and loose connective tissue in distal air spaces (alveolar spaces and bronchiolar lumen), although lung structure is preserved. Treatment tends to be steroid-responsive, though scarring may persist (Travis et al. 2013; King and Mortenson 1992). In our case, organizing pneumonia was held to be secondary and differentials included infections, malignancy, transtuzumab, paclitaxel or prior radiation. However the absence of any demonstrable pathogen and the occurrence of bilateral lesions outside the known extrathoracic radiation field precluded infections or radiation as a cause. The strong temporal association of hypersensitivity reactions with single agent transtuzumab and marked resolution of the disease process on stopping the drug, strongly suggests a role of transtuzumab in its aetiology. A probable causative or contributory role of malignancy in its aetiology, cannot be fully excluded.

Drug-induced infiltrative lung disease (DI-ILD) is the most common form of anti-neoplastic agent-induced respiratory disease. Its spectrum includes non-specific interstitial pneumonia, eosinophilic pneumonia, hypersensitivity pneumonitis, pulmonary fibrosis, or organizing pneumonia (Travis et al. 2013; King and Mortenson 1992).

The association of malignancy with BOOP is an infrequently described clinical entity and mimics various respiratory conditions. In the largest retrospective study describing this phenomenon, 43 patients with an underlying diagnosis of cancer were found on lung biopsy to have BOOP as an isolated entity. All patients had received therapy including chemotherapy (71%) or radiotherapy 9(21%), only 7 had surgery alone. Two patients who had received extra-thoracic radiation developed BOOP 2–3 years after treatment, while others who received thoracic radiation developed BOOP ipsilateral to the radiation port. One case of breast cancer was also reported. Patients with solid organ tumours were more likely to have nodular or mass like radiographic abnormalities (81%) rather than diffuse infiltrates (19%). The opposite pattern was observed in patients with hematologic malignancies (22 vs. 67%). Most patients recovered on steroids or macrolides but the disease was fatal in 3 patients with haematological malignancies (Mokhtari et al. 2002).

Transtuzumab is reasonably safe to use and only about 0.3% patients have serious reactions with features of anaphylaxis or bronchospasm, usually occurring within 2.5 h of drug administration (Cook-Burns 2001; Vahid and Mehrotra 2006).

Trastuzumab-associated pneumonitis is a rare, potentially fatal side effect, presenting as hypoxemia, dyspnoea and respiratory failure. Common radiological findings are interstitial infiltrates with a ground-glass appearance and patchy foci of airspace consolidation. In the trial National Surgical Adjuvant Breast and Bowel Program (NSABP) B-31, of 2102 women enrolled, four patients in the trastuzumab group had interstitial pneumonitis, one of whom who died (Vahid and Mehrotra 2006; Perez et al. 2014). In the North Central Cancer Treatment Group (NCCTG) N9831 trial, of 1944 women, five patients in the trastuzumab group had grade 3+ pneumonitis or pulmonary infiltrates, one of whom who died (Perez et al. 2014; Pepels et al. 2009; Romond et al. 2005).

Only three cases of transtuzumab induced organizing pneumonitis have been described so far. One patient, 49 years old, developed it 6 weeks after starting transtuzumab and this resolved three months after its cessation. Another patient, 60 years old, developed it after 11 cycles and this too resolved three months after drug cessation (Radzikowska et al. 2003; Taus-García et al. 2010; Grudny et al. 2004).

Pneumonitis rates of 4% have been described with albumen bound paclitaxel in combination with gemcitabine, especially in patients with pancreatic cancer. However none of these episodes were Grade 3 or higher and were not fatal (Von Hoff et al. 2013).

Paclitaxel is also known to cause pneumonitis with an estimated frequency of 0.7%, more so in combination with gemcitabine or irinotecan. Dyspnea, cough, hypoxemia, and pulmonary infiltrates usually develop 1 week to 3 months into therapy. Severe pneumonitis and pulmonary fibrosis resulting in death have been described but mild cases tend to resolve spontaneously (Khan et al. 1997).

## Conclusions

Organizing pneumonias can occur in patients with metastatic cancers. Patients undergoing treatments for cancer with either chemotherapy or targeted treatments may develop drug induced organizing pneumonitis as a rare adverse reaction. Clinical and radiological features closely mimic infection or progressive malignancy: thus a high index of suspicion is needed for diagnosis. Failure to diagnose may lead to delays in treatment and translate in deleterious patient outcomes to patients.

## Abbreviations

mg: milligram
m: metre
BAL: Broncho-alveolar fluid lavage
CECT: contrast enhanced contrast tomography
TTF-1: thyroid transcription factor
MRI: magnetic resonance imaging
COP: cryptogenic organizing pneumonia
BOOP: bronchiolitis obliterans organizing pneumonia
IIP: idiopathic interstitial pneumonias
